# New Mode of Relieving Retention of Urine

**Published:** 1859-10

**Authors:** Langston Parker

**Affiliations:** Surgeon to Queen’s Hospital, Birmingham


*New Mode of Relieving Retention of Urine.* Langston Parker, Esq., Surgeon to Queen's Hospital, Birmingham. (*Brit. Med. Jour.*, May 21, 1859.)

Dr. Parker states that he has very recently succeeded, in two separate instances, in relieving retention of urine, in the following manner:

"A gentleman lately entered my consultation room in great pain from retention of urine. He had not passed water for many hours; the bladder was much distended. He stated that ineffectual efforts had been made to pass a catheter, during which operations he had lost a considerable quantity of blood. I attempted to relieve him by the catheter, but failed to do so; I tried instruments of various sizes and various curves, but could not succeed in passing one into the bladder. I then took a No. 2 wax bougie, and inserted a small portion of potassa fusa into the end of it, after the manner proposed by Mr. Whateley and practised by Mr. Wade, in the treatment of permanent stricture of the uretha. I well moulded the wax over all but the extreme point of the caustic, and passed it rapidly down to the point of obstruction; by pressing against this for a short time it yielded, and I had the satisfaction of finding the bougie easily enter the bladder. I directed the patient to strain as I withdrew the instrument; a stream of urine followed, and the bladder was emptied. The retention did not again occur, and very little irritation accompanied or followed the proceeding. On the next day, the patient made water freely, but in a small stream.

"The second case was very similar. The patient had travelled some distance by rail. The bladder was much distended, the symptoms urgent, and a catheter could not be made to enter the bladder. A small wax bougie was armed as in the last case, passed down to the stricture, and firmly pressed against it. It yielded very quickly; the instrument entered the bladder, and a stream of urine followed its withdrawal. The patient had a second attack of retention two days afterwards, which was completely relieved in the same manner.

"A modification of this plan might be attempted by inserting a small piece of potassa fusa into the extreme point of a small gum elastic catheter, and using it without the stilette. I am sanguine enough to hope that many cases of retention of urine might be easily and quickly relieved by the simple means I have suggested, and more formidable and dangerous operations thus frequently avoided."—*Char. Med. Journal.*

